# Comparing tariff and medical assistant assigned causes of death from verbal autopsy interviews in Matlab, Bangladesh: implications for a health and demographic surveillance system

**DOI:** 10.1186/s12963-018-0169-1

**Published:** 2018-06-27

**Authors:** Riley H. Hazard, Nurul Alam, Hafizur Rahman Chowdhury, Tim Adair, Saidul Alam, Peter Kim Streatfield, Ian Douglas Riley, Alan D. Lopez

**Affiliations:** 1 School of Population and Global Health, University of Melbourne, Parkville, VIC Australia; 2 International Centre for Diarrhoeal Disease Research, Dhaka, Bangladesh

**Keywords:** Bangladesh, Verbal autopsy, Cause of death, Medical assistant, Tariff, Vital registration system

## Abstract

**Background:**

Deaths in developing countries often occur outside health facilities, making it extremely difficult to gather reliable cause of death (COD) information. Automated COD assignment using a verbal autopsy instrument (VAI) has been proposed as a reliable and cost-effective alternative to traditional physician-certified verbal autopsy, but its performance is still being evaluated. The purpose of this study was to compare the similarity of diagnosis by Medical Assistants (MA) in the Matlab Health and Demographic Surveillance System (HDSS) with the SmartVA Analyze 1.2 (Tariff 2.0) diagnosis.

**Methods:**

This study took place between January 2011 and April 2014 in Matlab, Bangladesh. MA with 3 years of medical training assigned COD to Matlab residents by reviewing the information collected using the Population Health Metrics Research Consortium (PHMRC) long-form VAI. Smart VA Analyze 1.2 automatically assigned COD using the same questionnaire. COD agreement and cause-specific mortality fractions (CSMFs) were compared for MA and Tariff.

**Results:**

Of the 4969 verbal autopsy cases reviewed, 4328 were adults, 296 were children, and 345 were neonates. Cohen’s kappa was 0.38 (0.36, 0.40) for adults, 0.43 (0.38, 0.49) for children, and 0.27 (0.22, 0.33) for neonates. For adults, the top two COD for MA were stroke (29.6%) and ischemic heart diseases (IHD) (14.2%) and for Tariff these were stroke (32.0%) and IHD (14.0%). For children, the top two COD for MA were drowning (33.5%) and pneumonia (13.2%) and for Tariff these were also drowning (36.8%) and pneumonia (12.4%). For neonates, the top two COD for MA were birth asphyxia (41.2%) and meningitis/sepsis (22.3%) and for Tariff these were birth asphyxia (37.0%) and preterm delivery (30.9%).

**Conclusion:**

The CSMFs for Tariff and MA showed very close agreement across all age categories but some differences were observed for neonate preterm delivery and meningitis/sepsis. Given the known advantages of automated methods over physician certified verbal autopsy, the SmartVA software, incorporating the shortened VAI questionnaire and Tariff 2.0, could serve as a cost-effective alternative to Matlab MA to routinely collect and analyze verbal autopsy data in a HDSS to generate essential population level COD data for planning.

**Electronic supplementary material:**

The online version of this article (10.1186/s12963-018-0169-1) contains supplementary material, which is available to authorized users.

## Background

Reliable information of cause of death is essential to inform health policy and planning [1]. Accurate and timely knowledge of the levels and trends of cause of death in a population provides health planners with the critical insight necessary to judge the priority for, and effectiveness of, current health programs as well as the evidence to address emerging health problems. However, vital registration (VR) systems that record causes of death are often of poor quality [2]. Developing countries typically only register about 25% of deaths; in the least developed countries this figure is only around 5–10% [3, 4].

Autopsy and/or accurate medical certification of cause of death by physicians are the “gold standards” for determining cause of death (COD). However, the majority of deaths in developing countries occur outside health facilities, limiting the scope of medical certification of cause of death [5, 6]. Verbal autopsy (VA) is a practical method for collecting illness-related symptoms and information from a close relative of the deceased and interpreting the interview data to assign a most probable underlying COD for a non-facility death. VA interviews have historically been reviewed by a physician to do this. This physician review method, however, suffers from a number of disadvantages: 1) cost, 2) non-standardized and hence incomparability of diagnostic procedures due to differences in the ways physicians interpret VAs, 3) the burden on physicians to review questionnaires and assign the COD which detracts them from delivering health services, and 4) poor agreement of assigned COD between certifying physicians. Consequently, faster, cheaper and standardized automated VA diagnostic methods have been developed to ascertain the COD in these settings [7, 8]. VA methods can now be used to routinely assign COD in VR systems due to major advances in ease of use and accuracy [9]. Indeed, the World Health Organization (WHO) has called for wider use of VA as a standardized method for determining population COD statistics [10].

Verbal autopsy instruments (VAI) utilize questionnaires conducted by trained staff to obtain retrospective information about the deceased’s signs, symptoms, and events surrounding the death. Current questionnaires are recorded on paper or Android tablets and sent to research centers to be collected and analyzed [11]. A number of computer methods have been applied to diagnose VA data, including Tariff and InterVA [12, 13]. In a large comparative and validation study, Tariff emerged as the most transparent and accurate VA method [9]. The Population Health Metrics and Research Consortium (PHMRC) VAI was recently shortened by 50% to reduce the time and resources required for interview, without a significant drop in performance [14]. This shortened VAI is suitable to apply in a routine mortality surveillance system using Smart VA Analyze 1.2 software (Tariff 2.0) [11, 15]. Tariff 2.0 is freely available from the Institute for Health Metrics and Evaluation (IHME) [16].

VAI have produced reliable cause-specific mortality fractions (CSMFs) in some settings [9]. However, many surveillance and research project sites prefer the critical cultural and region-specific insights of medical staff and train them to assign a COD by interpreting VA interviews. Trained medical assistants (MA) have been engaged as an alternative to physicians in determining COD. Their performance compared with physicians in diagnosing VAs is equivocal. In rural Bangladesh, no significant difference was found in the COD assignment for neonates between physicians and medical assistants with 3 years of training [17]. Conversely, a study based on data from Guatemala, Pakistan, Zambia, and the Democratic Republic of the Congo, found significant differences between physicians and non-physician COD assignment [18]. Nonetheless, medical assistants have longstanding relationships with the communities they work in as well as close contact with physicians in hospitals who conduct the medical certification of hospital deaths. Thus, MA should be a reliable mechanism to diagnose VAs, although this function takes them away from health care delivery duties. It is thus of interest to know whether automated VA methods can yield similar CODs information as the more expensive, time consuming, and variable experience with MA.

The International Centre for Diarrhoeal Disease Research, Bangladesh (icddr,b) maintains a comprehensive Health and Demographic Surveillance System (HDSS) in a rural area in Bangladesh. Since 2003, the HDSS has been using a structured VA questionnaire based on the recommendations of WHO to collect COD data [19]. This paper investigates the diagnostic agreement between MA and an automated VA diagnostic method, Tariff 2.0, in assigning COD from VA interviews in the Matlab HDSS.

## Methods

The study took place in Matlab, Bangladesh, an eastern Bangladesh sub-district where icddr,b maintains a HDSS. Matlab is located about 55 km southeast of the Bangladesh capital of Dhaka and has a population of about 225,000. In Matlab there has been surveillance for vital events since 1966; it is reasonably representative of the social and economic characteristics of the rural hospital catchment areas. Matlab HDSS employs Senior Health Research supervisors (SHRS) to conduct interviews of the families and caregivers of the deceased using the Matlab Verbal Autopsy Instrument, adjusted to include the long-form PHMRC VAI question items. SHRS conducted 4969 VA interviews on deaths which occurred during the study period, January 2011 to April 2014.

VA data and open-ended responses were translated into English, recorded on paper and manually entered into a project database. The data were then exported to an IHME cloud address. These VA interview data were prepared for PHMRC shortened questionnaires and analyzed by Smart VA Analyze 1.2 software (Tariff 2.0). Malaria and HIV were excluded as a COD because malaria and HIV are known to be rare causes of death in Matlab. Health care experience variables were used in the Tariff analysis to capture information that respondents knew about arising from the decedent’s contact with health care services [12]. MA reviewed the interview data, assigned a COD, and selected an ICD-10 code for each underlying COD. These codes were translated to a text COD using an ICD-10-to-VA cause list by age group (Additional file 1). Additional file 1 mirrors the 32 cause list that is used for the application of Tariff 2.0 with the addition of “other cancers” COD to the adult age group [15].

Cause-specific mortality fractions (CSMFs) for Tariff and MA were calculated for each age category: adult (12 years and above); child (29 days to 11 years); and neonate (less than 29 days). The level of agreement between Tariff and MA for each age group was quantified using Cohen’s kappa and between each cause using the chi-squared test at the 0.05 significance level. All causes of death outside of the top 15 most frequent causes in each age group were collapsed into the: COD Outside the Top 15″ category. Tariff COD was compared to that of MA both before and after the undetermined COD was reallocated using standard Global Burden of Disease (GBD) algorithms [15] Kappa agreement was only calculated prior to reallocation since deaths were reallocated at the population level, not individually.

## Results

The median age at death was 69 years (SD = 27.1, IQR = 26). Of the 4969 verbal autopsy cases reviewed, 4328 were adults, 296 were children, and 345 were neonates. Cohen’s kappa was 0.38 (0.36, 0.40) for adults, 0.43 (0.38, 0.49) for children, and 0.27 (0.22, 0.33) for neonates. Adult, children, and neonate CSMFs from MA and Tariff COD predictions showed close agreement (Tables 1, 2 and 3, Additional files 2, and 3).

**Table 1:** Cause-specific mortality fractions for Medical Assistants and Tariff for Adults (*n* = 4328)

Medical Assistants	CSMF	Tariff reallocated	CSMF
Stroke	29.6	Stroke	32.0
Ischemic Heart Disease	14.2	Ischemic Heart Disease	14.0
Other Cardiovascular Diseases	8.2	Chronic Respiratory	11.0
Other NCD	7.4	Diabetes	6.0
Chronic Respiratory	6.7	Other NCD	5.0
Other Cancers	6.4	Cirrhosis	4.0
Other Infectious Diseases	3.6	Lung Cancer	3.0
Diabetes	2.3	Falls	3.0
Lung Cancer	1.6	TB	2.0
Falls	1.5	Diarrhea/Dysentery	2.0
Diarrhea/Dysentery	1.4	Road Traffic	2.0
Cirrhosis	1.4	Other Injuries	2.0
Other Injuries	1.2	Drowning	1.0
COD Outside the Top 15	10.2	Other Infectious Diseases	1.0
Undetermined	5.6	COD Outside the Top 15	12.0

*CSMF* cause-specific mortality fraction, *COD* cause of death, *NCD* non-communicable diseases

**Table 2:** Cause-specific mortality fractions for Medical Assistants and Tariff for Children (*n* = 296)

Medical Assistants	CSMF	Tariff reallocated	CSMF
Drowning	33.5	Drowning	36.8
Pneumonia	13.2	Pneumonia	12.4
Sepsis	4.7	Other Cardiovascular Diseases	9.9
Other Cancers	4.1	Other Cancers	8
Diarrhea/Dysentery	2.7	Other Infectious Diseases	7.1
Other Digestive Diseases	2.4	Other Digestive Diseases	4.2
Meningitis	1.7	Diarrhea/Dysentery	3.3
Other Cardiovascular Diseases	1.7	Meningitis	3
Encephalitis	1	Hemorrhagic fever	2.9
Other Infectious Diseases	0.7	Road Traffic	1.7
Road Traffic	0.7	Encephalitis	1.7
Bite of Venomous Animal	0.3	Fires	0.8
Other Child Causes of Death	24.7	Violent Death	0.8
COD Outside the Top 15	1.4	Other Child Causes of Death	4.9
Undetermined	7.8	COD Outside the Top 15	3.2

*CSMF* cause-specific mortality fraction, *COD* cause of death

**Table 3:** Cause-specific mortality fractions for Medical Assistants and Tariff for Neonates (*n* = 345)

Medical Assistants	CSMF	Tariff reallocated	CSMF
Birth asphyxia	41.2	Birth asphyxia	37
Meningitis/Sepsis	22.3	Preterm Delivery	30.9
Preterm Delivery	8.1	Pneumonia	17.1
Congenital malformation	6.7	Meningitis/Sepsis	7.5
Pneumonia	2.9	Congenital malformation	6.9
Stillbirth	0	Stillbirth	0.4
Undetermined	18.8	Undetermined	0

*CSMF* cause-specific mortality fraction

For adults, MA estimated more people died from other cardiovascular diseases and other cancers when compared with Tariff. MA and Tariff COD estimates for children showed MA attributing more deaths to sepsis while Tariff assigned more deaths to other cardiovascular diseases and other infectious diseases. For neonates, MA assigned more deaths to meningitis/sepsis and Tariff assigned more deaths to preterm delivery and pneumonia.

The top three causes of death for adults estimated by MA were stroke (29.6%), IHD (14.2%), and other cardiovascular diseases (8.2%) while for Tariff they were stroke (32.0%), IHD (14.0%), and chronic respiratory (11.0%) for the same population (Table 1). The top two causes of death for children estimated by MA were drowning (33.5%) and pneumonia (13.2%), identical to Tariff (drowning (36.8%); pneumonia (12.4%) (Table 2). The top two causes of death for neonates estimated by MA were birth asphyxia (41.2%) and meningitis/sepsis (22.3%); for Tariff they were birth asphyxia (37.0%) and preterm delivery (30.9%) (Table 3). Prior to reallocation, Tariff assigned 18.5, 24.0, and 22.6% to undetermined COD for adults, children, and neonates, respectively, which were then reallocated across causes using standard algorithms, compared to 5–18% for MA (Additional file 2) [15].

**Fig. 1:**
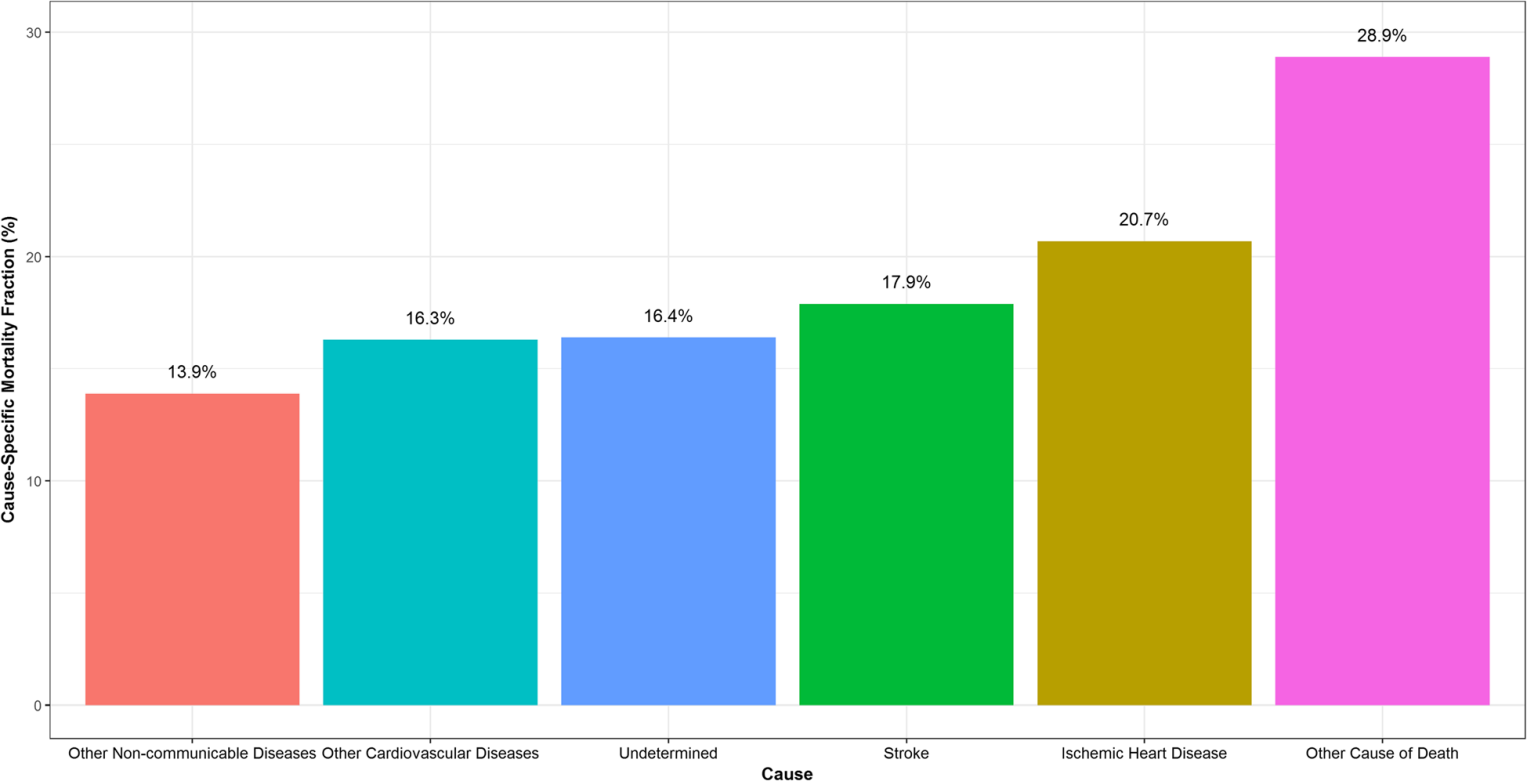
Distribution of Medical Assistant Assigned Cause of Death within 799 cases (19%) of Tariff Assigned Adult Undetermined Cause of Death

**Fig. 2:**
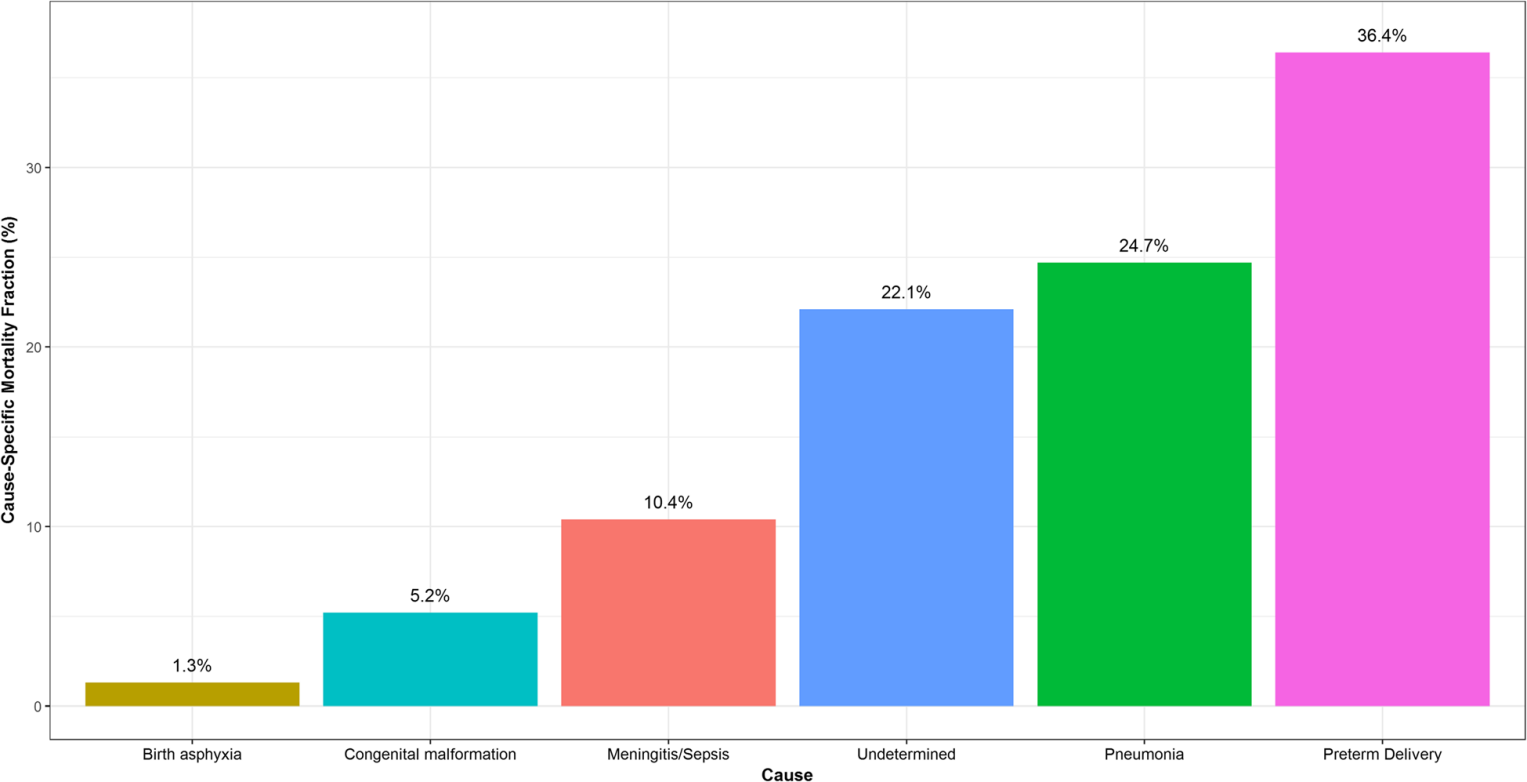
Distribution of Tariff Assigned Cause of Death within 77 cases (22%) of Medical Assistant Assigned Neonate Meningitis/Sepsis Cause of Death

**Fig. 3:**
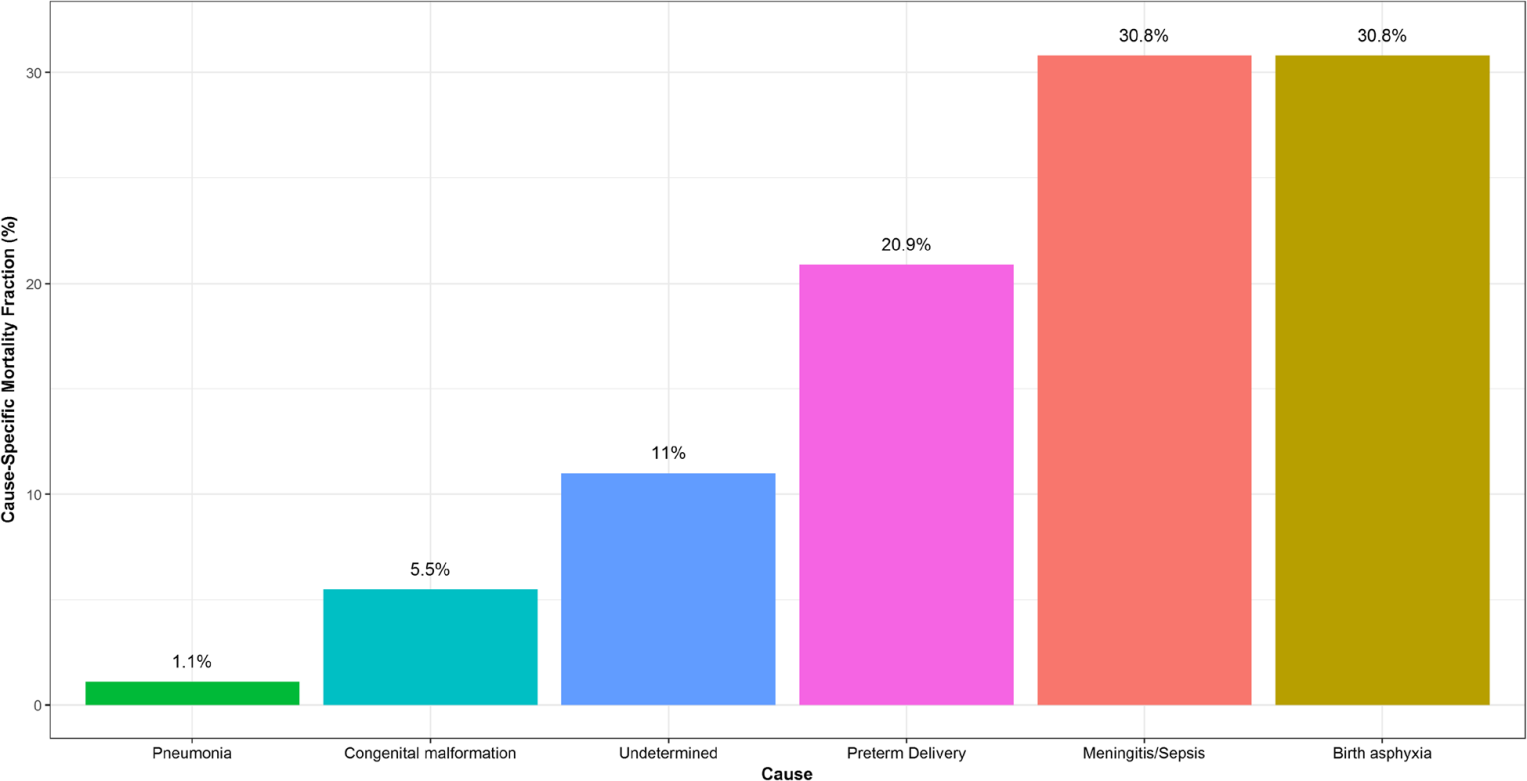
Distribution of Medical Assistant Assigned Cause of Death within 91 cases (26%) of Tariff Assigned Neonate Preterm Delivery Cause of Death

Figures 1, 2 and 3 explores the important diagnostic differences between Tariff and MA for those causes where there were large differences in the CSMFs. Of the 19% of adult deaths that Tariff assigned an undetermined COD, MA assigned 21% to IHD and 18% to stroke (Fig. 1). Of the 22% of neonate deaths attributed to meningitis/sepsis by MA, 36% were attributed to preterm delivery and 25% were attributed to pneumonia by Tariff (Fig. 2). Of the 26% of neonate deaths attributed to preterm delivery by Tariff, 31% were attributed to meningitis/sepsis and 31% were attributed to birth asphyxia by MA (Fig. 3).

## Discussion

This study compared the COD assignment of Bangladeshi MA to that of an automated VA method (Tariff) using the same PHMRC VAI. MA and Tariff CSMFs showed close agreement for all age groups, with children and adults showing very close agreement compared to the moderate agreement of neonates. The most frequent causes of death were in close agreement for all age groups, but some discrepancies existed for neonate meningitis/sepsis and preterm delivery. Tariff also assigned a moderate portion of deaths to an undetermined COD for all age groups.

Without a “gold standard” COD for all of these deaths, we were unable to determine the comparative COD performance of MA and Tariff, as has been done elsewhere [9]. However, the moderate Kappa scores suggest potential misclassifications between MA and Tariff. Tariff also assigned an intermediate portion of deaths to an undetermined COD that MA assigned to IHD and stroke. This may well reflect the quality of the interview, given how strongly dependent the method is on defining symptom-cause relationships in the data. One would expect the fraction of undetermined deaths using Tariff to diminish over time as the skills and expertise of interviewers increased. Preterm delivery, meningitis/sepsis, birth asphyxia, and pneumonia were frequently mixed, suggesting that either MA or Tariff had trouble distinguishing between these causes of death.

The MA who assigned COD based on the PHMRC VAI in this study resemble the physician-certified verbal autopsy (PCVA) method of assigning COD. In PCVA, hospital physicians review VAI data, but there is often little consistency in the methods of attributing a COD across physicians, even for the same death cases in different populations. In fact, PCVA has been shown to perform poorly relative to Tariff and other algorithms [9, 12]. MA in this study, however, offer a more consistent approach to assigning COD since Matlab has served as demographic surveillance site since 1966 and MA have a long history of assigning COD based on VAI. Nonetheless, without a gold standard comparison, it is difficult to know whether MA also displayed the same diagnostic errors as physicians in diagnosing the COD [20].

Though MA and Tariff showed close COD agreement, Tariff assigned a larger proportion of deaths to an undetermined COD, similar to applications of Tariff elsewhere. Following reallocation of the undetermined COD using the Tariff reallocation algorithm, based in part on GBD estimates for Bangladesh, the agreement was even closer [15]. For public health purposes, the consistency of diagnosis between MA and Tariff suggests that Smart VA Analyze, which incorporates the Tariff method, provides at least as reliable information on causes of death to guide public health priority in Bangladesh as MAs, at a fraction of the cost. The improved agreement when applying the Tariff reallocation algorithm to indeterminate causes suggests that reallocation of indeterminate cases should be routinely applied when interpreting the output of automated diagnostic methods such as Tariff in civil registration and vital systems.

Both MA and Tariff showed close similarity in ranking the most frequent causes of death for all age groups. For adults, non-communicable diseases, particularly stroke, IHD, and cardiovascular diseases were identified by both sources as being of upmost public health importance. For children, drowning and pneumonia remain public health prioties, as is birth asphyxia for neonates. There is little reliable Bangladeshi COD statistics from the CR system, but recent studies suggest that these causes are likely to be among the leading causes of death in the country [21, 22].

Without an adequate number of “gold standard” cases for which the true COD was known with reasonable certainty, it is difficult to judge which of the two methods performs better. Due to the relatively small number of in-hospital deaths (*n* = 117), there was very substantial uncertainty in the accuracy of chance corrected concordance (CCC) and chance corrected cause-specific morality fractions (CCCSMFs) statistics for the two methods [23]. These metrics are the recommended standards on which to assess diagnostic accuracy but were too uncertain to be of any use in this study.

MA also had additional sources of bias in estimating COD. First, MA had the ability to contact family members of the deceased when they thought there was insufficient information in the VA questionnaire, so some MA COD estimations may have been based on more information than that available to Tariff. This bias is similar to the improved diagnostic performance of physicians when they have information available about household recall of health care experience in PCVA [20]. Second, the VA questionnaire asked the family whether they had a death certificate and whether the interviewer could view it. 10% of the families had a death certificate and 5% provided the staff member with the certificate. VA questionnaires for these families may have been influenced by the fact that the families knew the probable COD from contact with medical services.

Comparing MA and Tariff COD estimation required mapping MA ICD-10 codes to Tariff COD groupings. IHME and WHO have released mappings of VA COD to ICD-10 ranges, but mapping from VA to ICD-10 is not inclusive of all ICD-10 codes or consistent between IHME and WHO. When there were inconsistencies, we used our best adjustment to appropriately assign a VA cause group to an ICD-10 code (Additional file 1). In addition, our comparative assessment of Tariff and MA was focused on the primary or most probable underlying COD, but for many deaths, particularly at older ages, this may be difficult to diagnose reliably in the presence of multiple morbid conditions. Last, reallocation of indeterminate causes was only performed at the population level, so we were unable to calculate Cohen’s kappa for the comparison between MA and reallocated Tariff.

## Conclusions

The results of this study suggest that Tariff is likely to be a very useful and cost-effective alternative to current practice using MAs to generate COD information in rural regions of Bangladesh. However, more definitive evidence about the comparative diagnostic accuracy of the two approaches can only be generated by comparing diagnoses to some gold standard where the true COD is known. This would require a carefully designed validation study. MA do provide important cultural insights into assigning COD, but given the limited health resources in rural Bangladesh, MA time used for VAs might be more usefully deployed in providing medical care. The shortened PHMRC questionnaire with Smart VA Analyze (Tariff) offers valuable cost and time savings that may help improve mortality surveillance in developing countries, including Bangladesh.

## Additional files

Additional file 1: ICD-10 codes to text COD mapping. Three separate tables that provide mapping from ICD-10 codes to text causes of death for adults, children, and neonates. (DOCX 14 kb)

Additional file 2: Cause-specific mortality fractions for medical assistants, Tariff, and reallocated Tariff by age group. Three separate tables that provide cause-specific mortality fractions for medical assistants, Tariff, and reallocated Tariff for adults, children, and neonates. These tables differ from Tables 1, 2, and 3 because these include Tariff cause-specific mortality fractions prior to reallocation of the undetermined cause of death. (XLSX 14 kb)

Additional file 3: Cause-specific mortality fractions for medical assistants and reallocated Tariff by age group. Bar graphs that mirror Table 1 by comparing the cause-specific mortality fraction for medical assistants to reallocated Tariff with the addition of indicating statistical significance at the 0.05 significance level. (PNG 72 kb)

## Abbreviations

COD: Cause of death
CSMFs: Cause-specific mortality fractions
GBD: Global burden of disease
HDSS: Health and demographic surveillance system
ICD: International classification of diseases
icddr,b: International centre for diarrhoeal disease research, Bangladesh
IHD: Ischemic heart disease
IHME: Institute for health metrics and evaluation
MA: Medical assistant
PCVA: Physician-certified verbal autopsy
PHMRC: Population health metrics research consortium
SHRS: Senior health research supervisors
VA: Verbal autopsy
VAI: Verbal autopsy instrument
WHO: World Health Organization
