# Supplementary material for: Comparing tariff and medical assistant assigned causes of death from verbal autopsy interviews in Matlab, Bangladesh: implications for a health and demographic surveillance system
Source: Popul Health Metr. 2018 Jun 27;16:10. doi: 10.1186/s12963-018-0169-1 (PMC6020332; doi:10.1186/s12963-018-0169-1)
Supplement: Supplementary file 1 — ICD-10 codes to text COD mapping. Three separate tables that provide mapping from ICD-10 codes to text causes of death for adults, children, and neonates. (DOCX 14 kb) [file 12963_2018_169_MOESM1_ESM.docx]

| **Adult Text** | **Adult ICD-10 Code** |
| --- | --- |
| Diarrhea/Dysentery | A00-A09 |
| TB | A15-A19 |
| AIDS | B20-B24 |
| Malaria | B50-B54 |
| Other Infectious Diseases | A10-A14, A20-B19, B25-B49, B55-B99 |
| Esophageal Cancer | C15 |
| Stomach Cancer | C16 |
| Colorectal Cancer | C18-C21 |
| Lung Cancer | C34 |
| Breast Cancer | C50 |
| Cervical Cancer | C53 |
| Prostate Cancer | C61 |
| Leukemia/Lymphoma | C81-C85; C91-C96 |
| Other Cancers | C00-C14, C17, C22-C33, C35-C49, C51-C52, C54-C60, C62-C80, C86-C90, C97-D48 |
| Diabetes | E10-E14 |
| Other Cardiovascular Diseases | I00-I19 I26-I59, I70-I99 |
| Ischemic Heart Diseases | I20-I25 |
| Stroke | I60-I69 |
| Pneumonia | J10-J22,J85 |
| Chronic Respiratory diseases (COPD/Asthma) | J40-J46 |
| Cirrhosis | K70-K76 |
| Renal Failure | N17-N19 |
| Maternal | O00-O99 |
| Undetermined | R00-R99 |
| Road Traffic | V01-V89 |
| Falls | W00-W19 |
| Drowning | W65-W74 |
| Fires | X00-X19 |
| Bite of Venomous Animal | X20-X29 |
| Poisonings (accidental) | X40-X49 |
| Suicide (intentional self-harm) | X60-X84 |
| Homicide (assault) | X85-Y09 |
| Other Injuries | S00-T98, V90-V99, W20-W64, W75-W99, X30-X39, X50-X59, Y10-Y98 |
| Other Non-communicable Diseases | All other ICD-10 codes |

| **Child Text** | **Child ICD-10 Code** |
| --- | --- |
| Diarrhea/Dysentery | A00-A09 |
| Sepsis | A40-A41 |
| Hemorrhagic fever | A92-A99 |
| Measles | B05 |
| AIDS | B20-B24 |
| Malaria | B50-54 |
| Other Infectious Diseases | A10-A39, A42-A91, B00- B04, B06-B49, B55-B99 |
| Other Cancers | C00-D48 |
| Meningitis | G00-G03,A39,A87 |
| Encephalitis | G04;A83-A86 |
| Other Cardiovascular Diseases | I00-I99 |
| Pneumonia | J10-J22,J85 |
| Other Digestive Diseases | K00-K93 |
| Undetermined | R00-R99 |
| Road Traffic | V01-V89 |
| Falls | W00-W19 |
| Drowning | W65-W74 |
| Fires | X00-X19 |
| Bite of Venomous Animal | X20-X29 |
| Poisonings | X40-X49 |
| Violent Death | X85-Y09 |
| Other Defined Causes of Child Deaths | All other ICD-10 codes |

| **Neonate Text** | **Neonate ICD-10 Code** |
| --- | --- |
| Preterm Delivery | P05-P07 |
| Birth asphyxia | P20-P22 |
| Pneumonia | P23-P25,J10-J22 |
| Meningitis/Sepsis | P36, G00-G04, A39, A87 |
| Stillbirth | P95 |
| Congenital malformation | Q00-Q99 |
| Undetermined | All other ICD-10 codes |
