# Supplementary figures and images for: Comparing tariff and medical assistant assigned causes of death from verbal autopsy interviews in Matlab, Bangladesh: implications for a health and demographic surveillance system
Source: Popul Health Metr. 2018 Jun 27;16:10. doi: 10.1186/s12963-018-0169-1 (PMC6020332; doi:10.1186/s12963-018-0169-1)

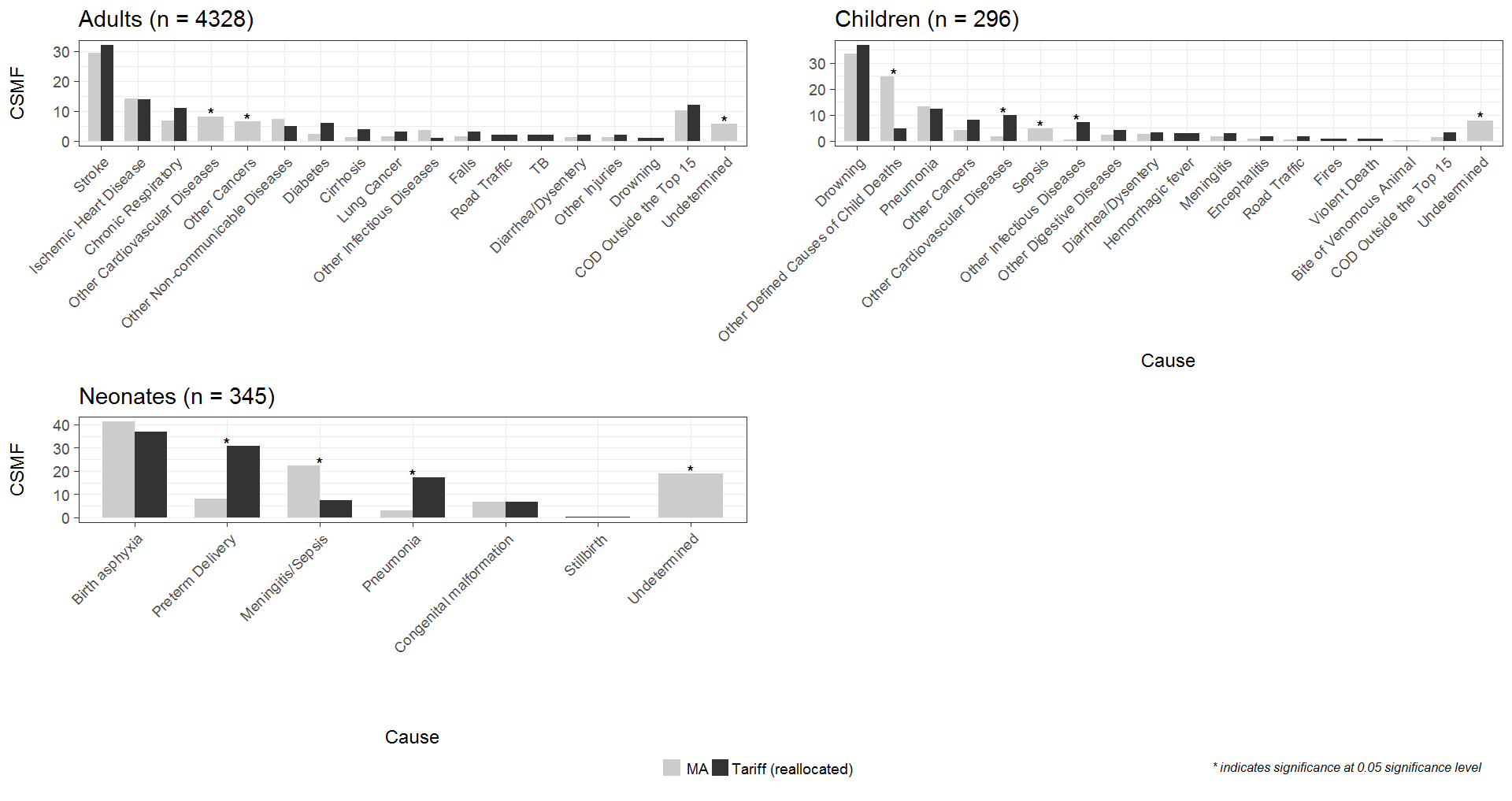

Supplement: Supplementary file 3 — Cause-specific mortality fractions for medical assistants and reallocated Tariff by age group. Bar graphs that mirror Table 1 by comparing the cause-specific mortality fraction for medical assistants to reallocated Tariff with the addition of indicating statistical significance at the 0.05 significance level. (PNG 72 kb) [file 12963_2018_169_MOESM3_ESM.png]
